# Supplementary material for: Survival Difference of Endometrial Cancer Patients with Peritoneal Metastasis Receiving Cytoreductive Surgery (CRS) with and without Hyperthermic Intraperitoneal Chemotherapy (HIPEC): A Systematic Review and Meta-Analysis
Source: Int J Mol Sci. 2024 Jul 8;25(13):7495. doi: 10.3390/ijms25137495 (PMC11242309; doi:10.3390/ijms25137495)
Supplement: Supplementary file 1 [file ijms-25-07495-s001.zip › ijms-3077233-supplementary.pdf]

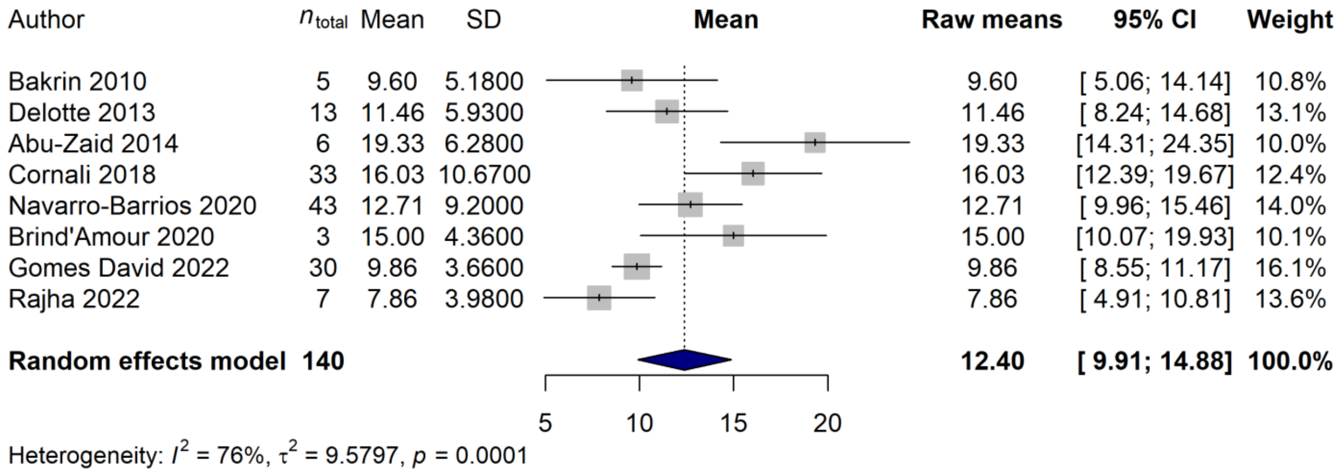

Figure S1. Mean peritoneal carcinomatosis index (PCI) retrieved from the investigated studies. All patients suffered from endometrial cancer with peritoneal metastasis, which was treated with cytoreductive surgery (CRS) with hyperthermic intraperitoneal chemotherapy (HIPEC). Whiskers, dotted line and blue diamond represents the raw mean and its 95% confidence interval (CI), the computed effect size, and the latter's CI, respectively.

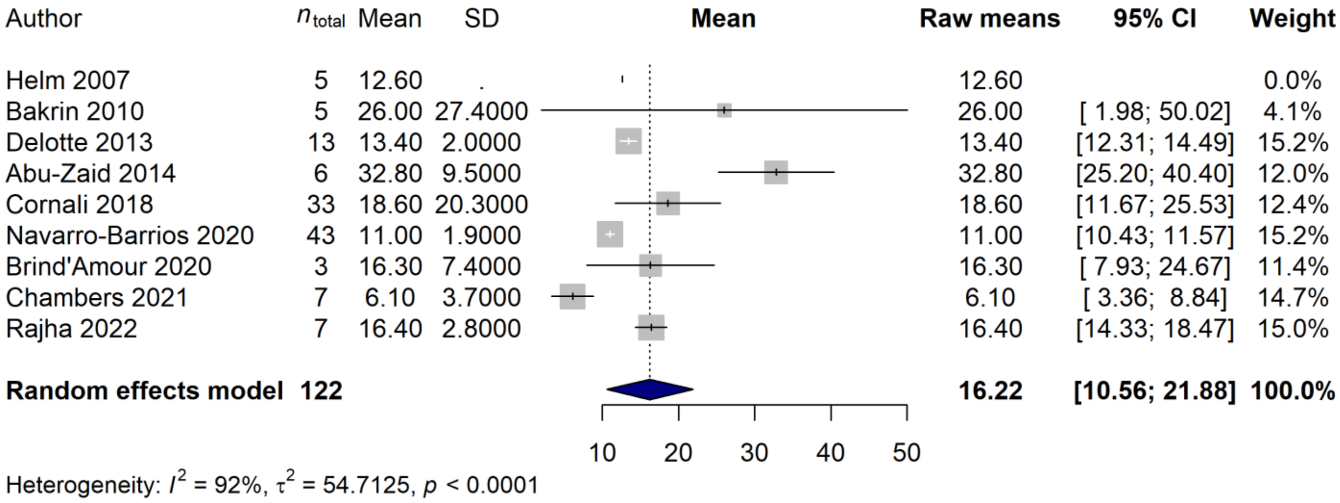

Figure S2. Mean hospital stay length (days) retrieved from the investigated studies. All patients suffered from endometrial cancer with peritoneal metastasis, which was treated with cytoreductive surgery (CRS) with hyperthermic intraperitoneal chemotherapy (HIPEC). Whiskers, dotted line and blue diamond represents the raw mean and its 95% confidence interval (CI), the computed effect size, and the latter's CI, respectively.

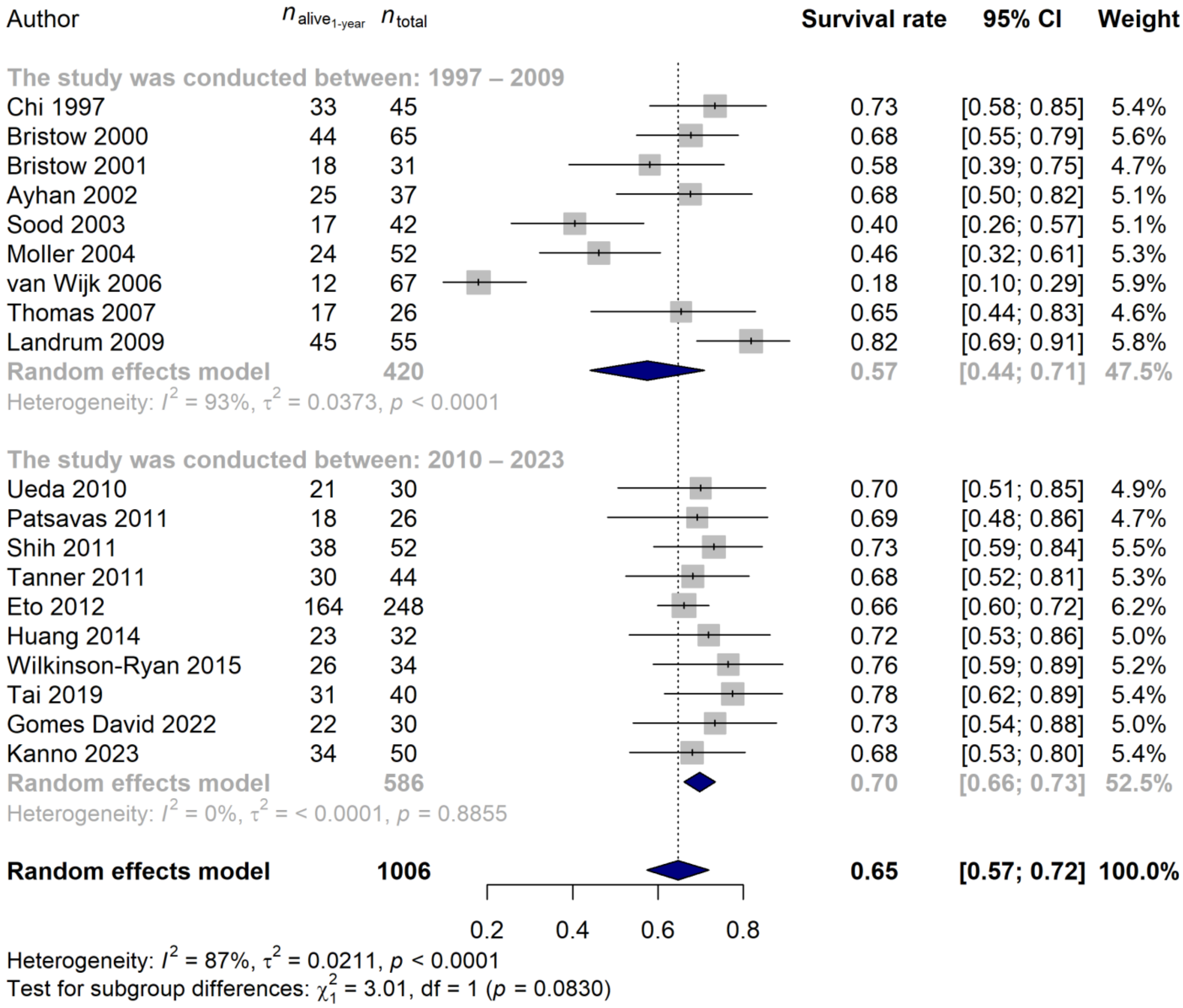

Figure S3. Comparison of the studies investigating 1-year survival rates, which were conducted before or after 2010. All patients suffered from endometrial cancer with peritoneal metastasis, which was treated with cytoreductive surgery (CRS) only. Whiskers, dotted line and blue diamond represents the 1-year survival rate and its 95% confidence interval (CI), the computed effect size, and the latter's CI, respectively.

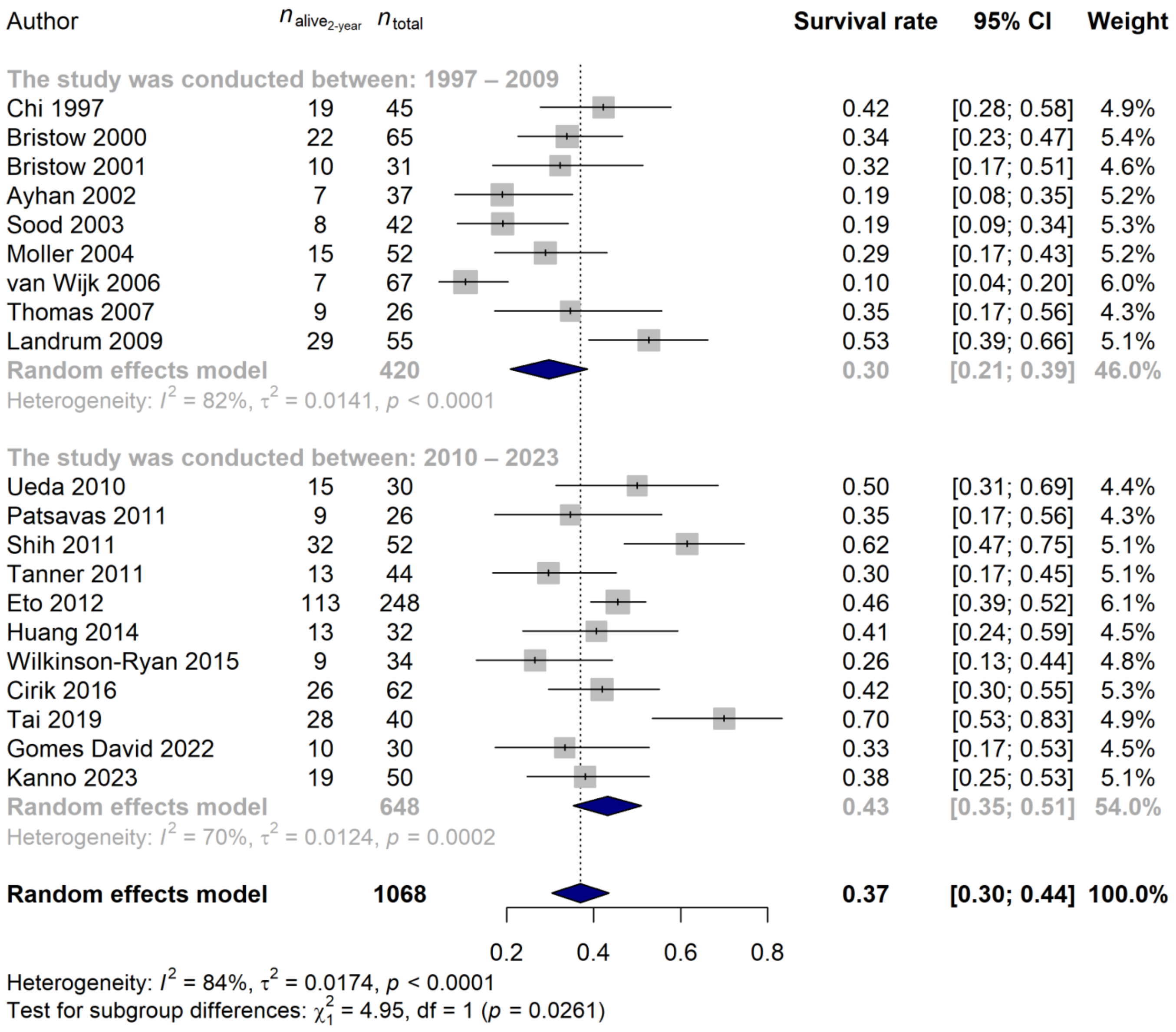

Figure S4. Comparison of the studies investigating 2-year survival rates, which were conducted before or after 2010. All patients suffered from endometrial cancer with peritoneal metastasis, which was treated with cytoreductive surgery (CRS) only. Whiskers, dotted line and blue diamond represents the 2-year survival rate and its 95% confidence interval (CI), the computed effect size, and the latter's CI, respectively.

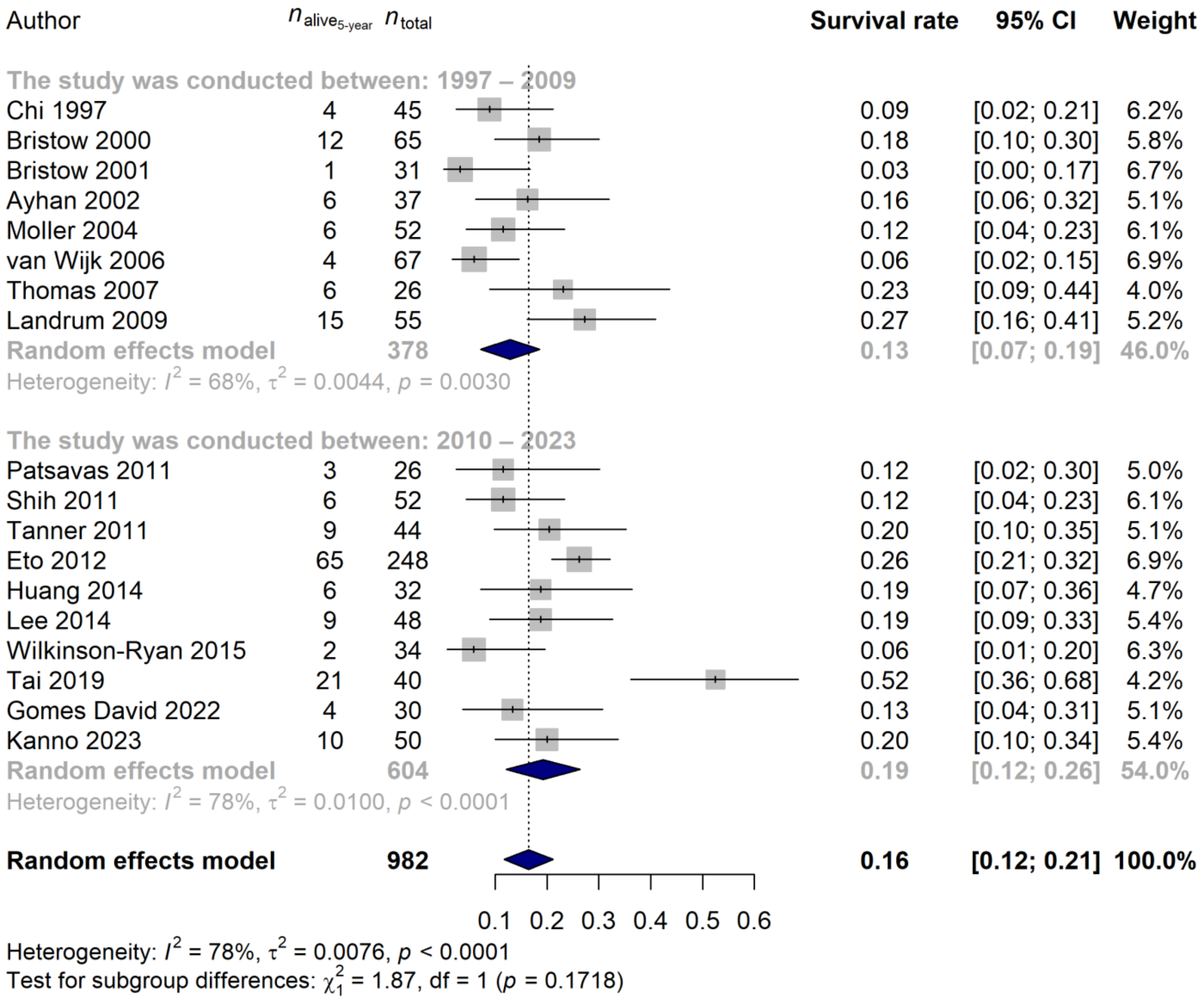

Figure S5. Comparison of the studies investigating 5-year survival rates, which were conducted before or after 2010. All patients suffered from endometrial cancer with peritoneal metastasis, which was treated with cytoreductive surgery (CRS) only. Whiskers, dotted line and blue diamond represents the 5-year survival rate and its 95% confidence interval (CI), the computed effect size, and the latter's CI, respectively.

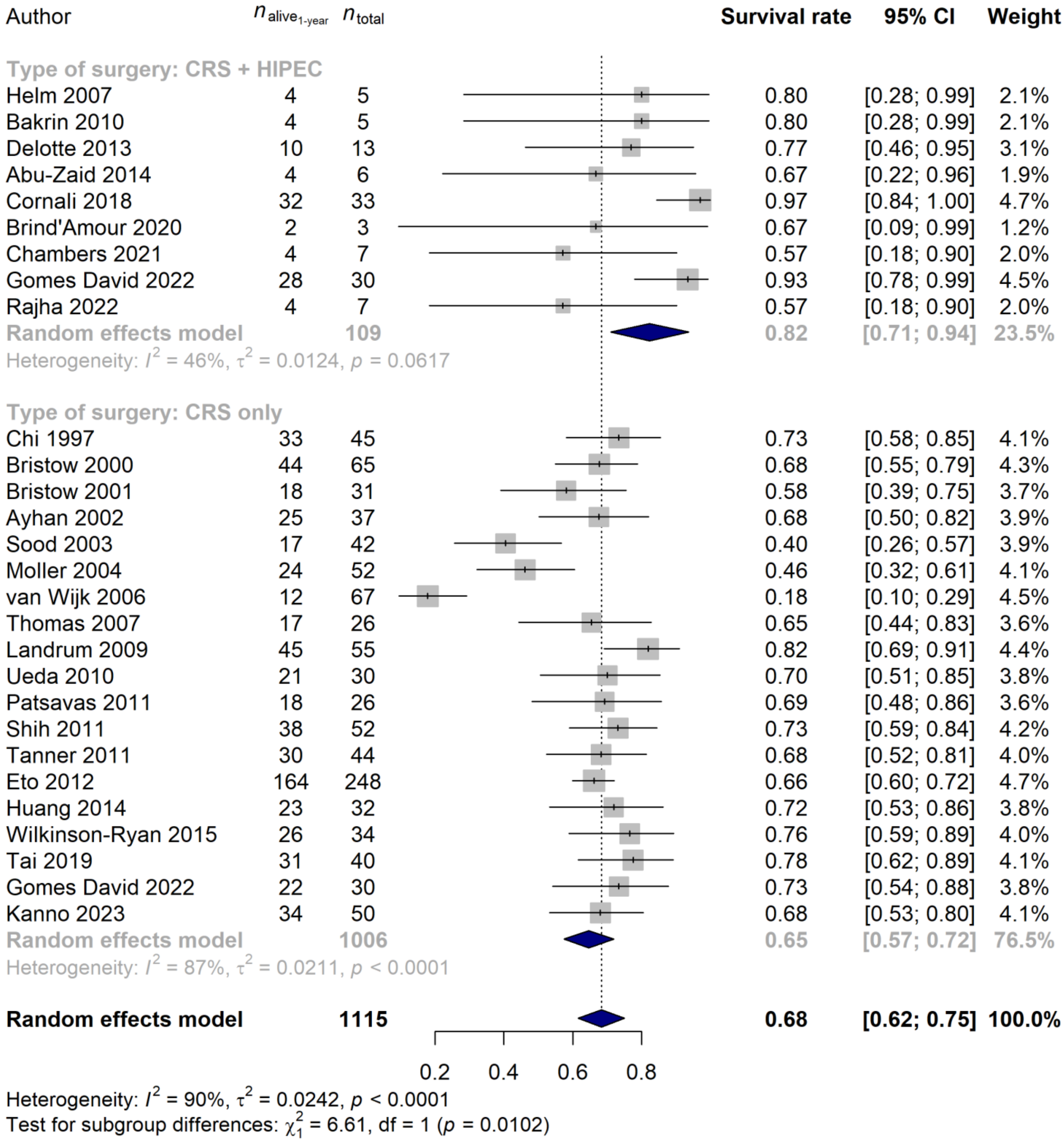

Figure S6. Comparison of the 1-year survival rates of patients suffering from endometrial cancer with peritoneal metastasis, which was treated with cytoreductive surgery (CRS) with or without hyperthermic intraperitoneal chemotherapy (HIPEC). A significantly higher 1-year survival rate is expected favoring CRS + HIPEC. Whiskers, dotted line and blue diamond represents the 1-year survival rate and its 95% confidence interval (CI), the computed effect size, and the latter's CI, respectively.

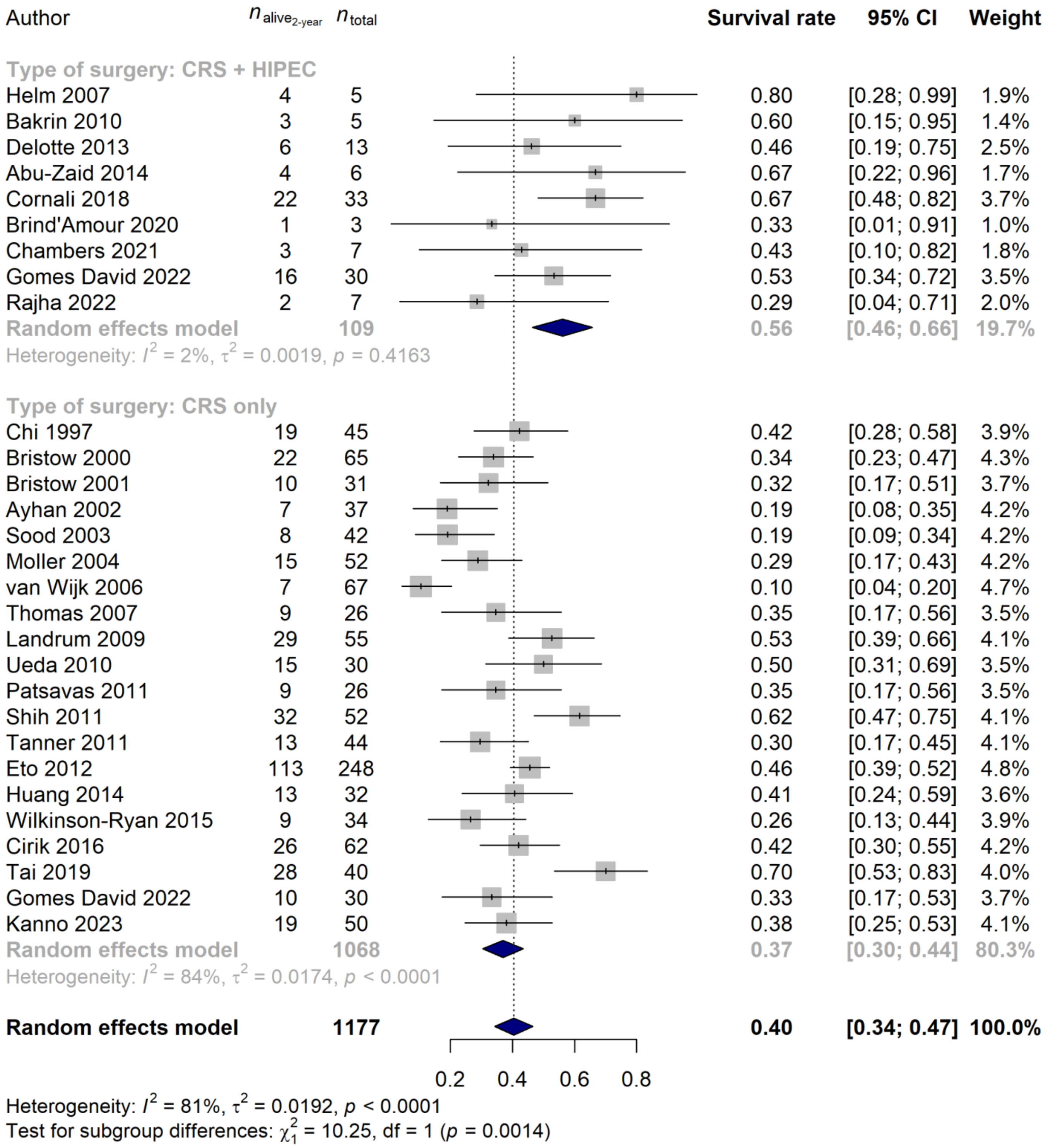

Figure S7. Comparison of the 2-year survival rates of patients suffering from endometrial cancer with peritoneal metastasis, which was treated with cytoreductive surgery (CRS) with or without hyperthermic intraperitoneal chemotherapy (HIPEC). A significantly higher 2-year survival rate is expected favoring CRS + HIPEC. Whiskers, dotted line and blue diamond represents the 2-year survival rate and its 95% confidence interval (CI), the computed effect size, and the latter's CI, respectively.

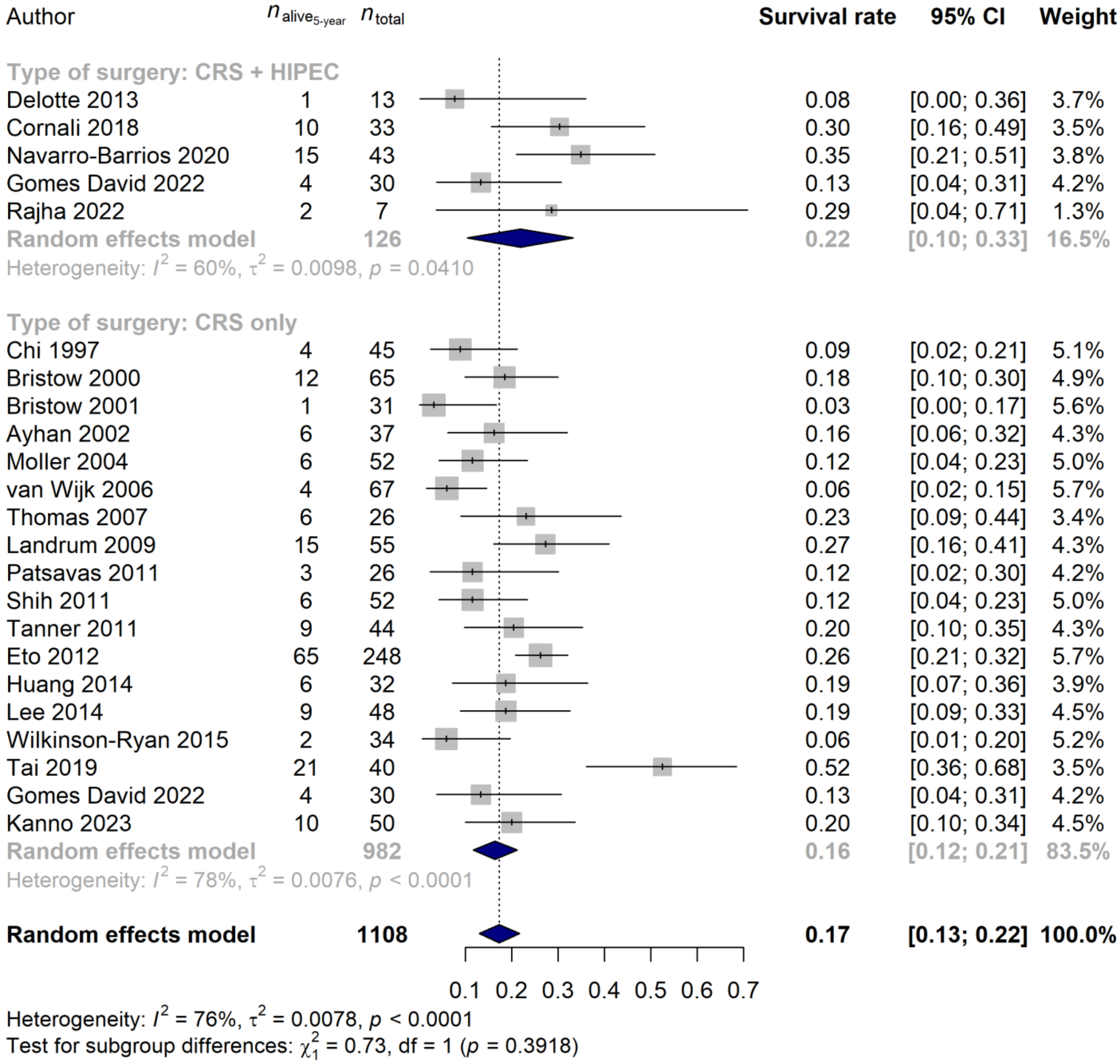

Figure S8. Comparison of the 5-year survival rates of patients suffering from endometrial cancer with peritoneal metastasis, which was treated with cytoreductive surgery (CRS) with or without hyperthermic intraperitoneal chemotherapy (HIPEC). No difference could be identified between the two treatment modalities. Whiskers, dotted line and blue diamond represents the 5-year survival rate and its 95% confidence interval (CI), the computed effect size, and the latter's CI, respectively.

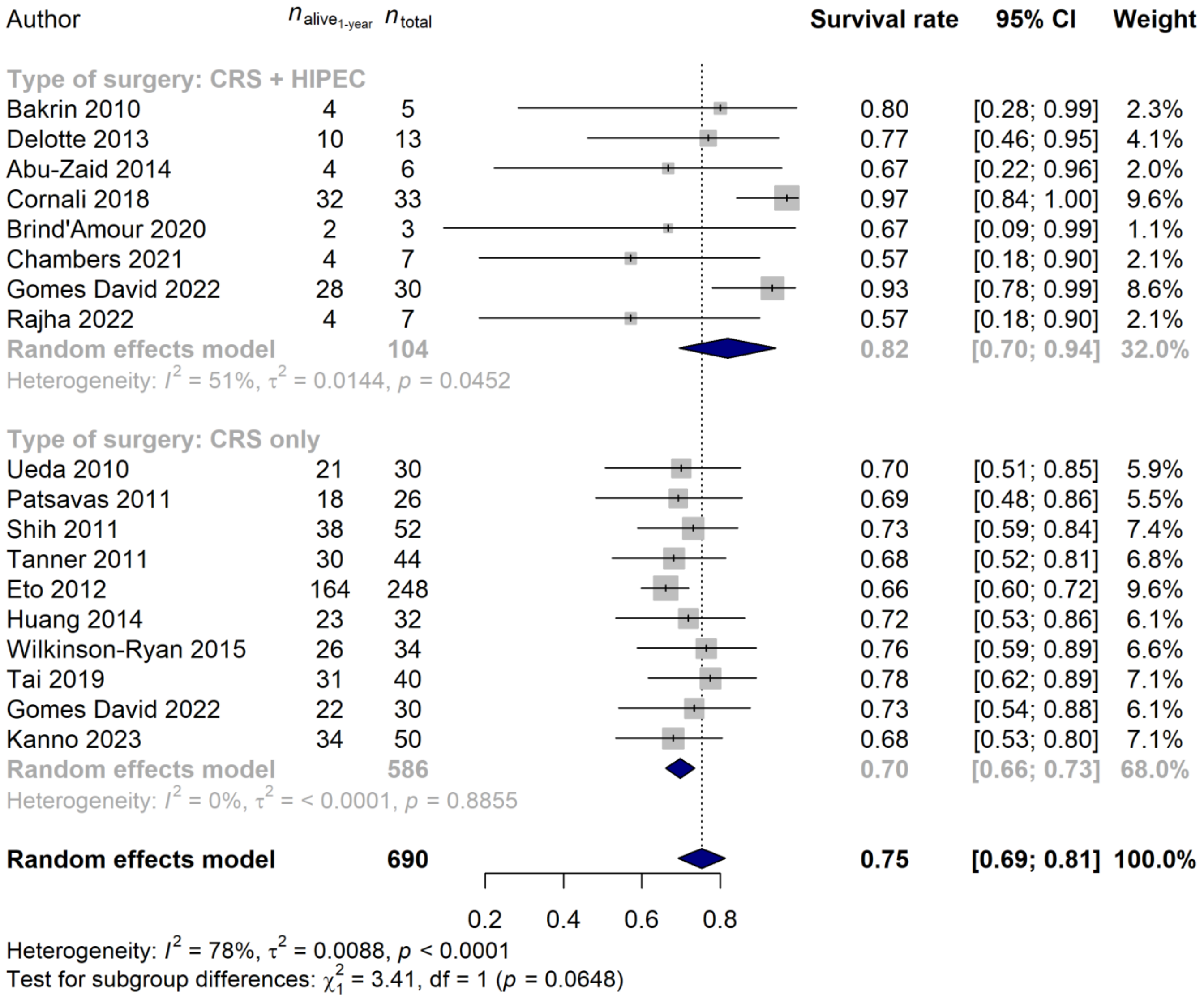

Figure S9. Comparison of the 1-year survival rates of the studies conducted after 2010, which have investigated patients suffering from endometrial cancer with peritoneal metastasis. Patients were treated with cytoreductive surgery (CRS) with or without hyperthermic intraperitoneal chemotherapy (HIPEC). A marginally higher 1-year survival rate is expected favoring CRS + HIPEC. Whiskers, dotted line and blue diamond represents the 1-year survival rate and its 95% confidence interval (CI), the computed effect size, and the latter's CI, respectively.

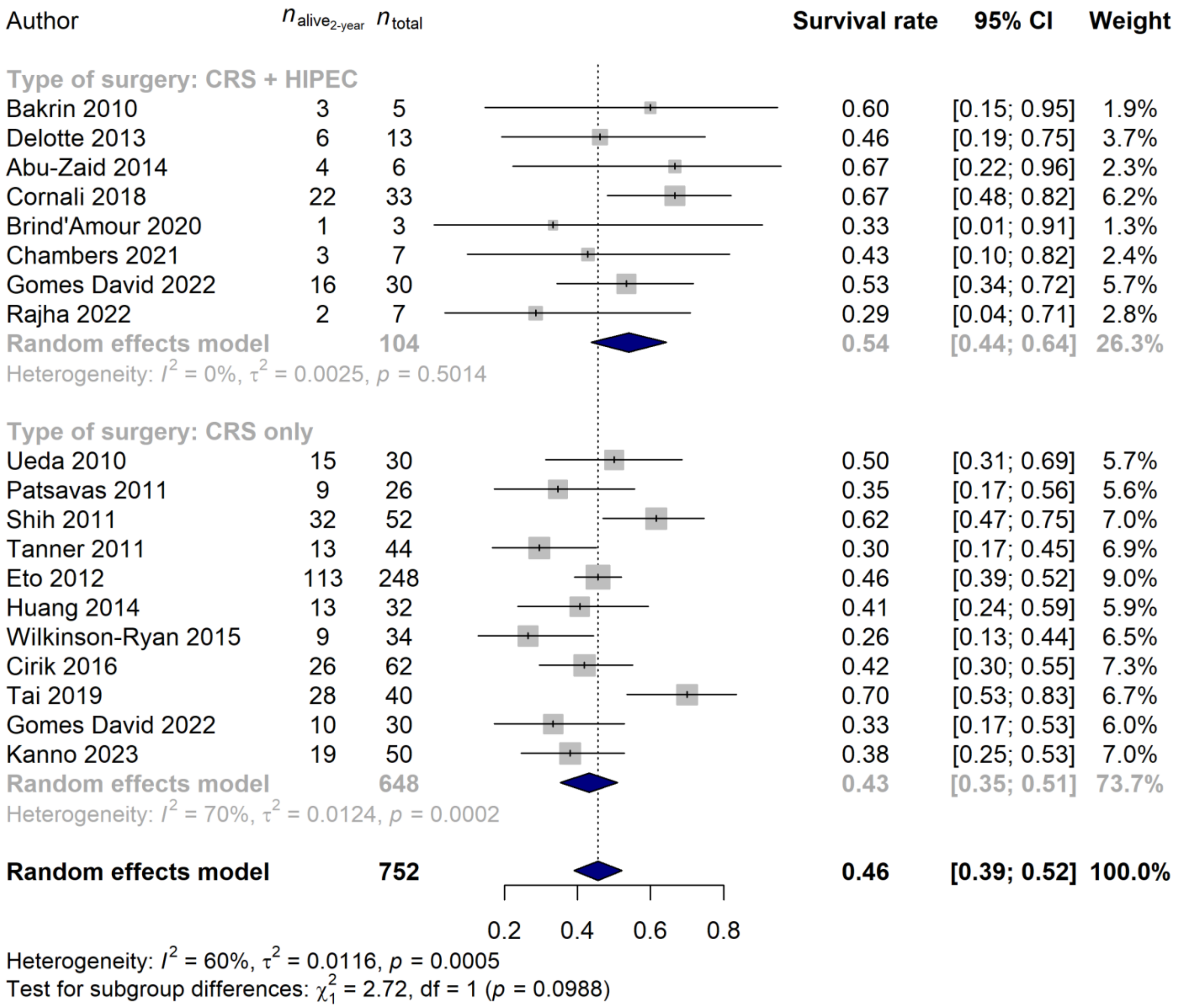

Figure S10. Comparison of the 2-year survival rates of the studies conducted after 2010, which have investigated patients suffering from endometrial cancer with peritoneal metastasis. Patients were treated with cytoreductive surgery (CRS) with or without hyperthermic intraperitoneal chemotherapy (HIPEC). A marginally higher 2-year survival rate is expected favoring CRS + HIPEC. Whiskers, dotted line and blue diamond represents the 2-year survival rate and its 95% confidence interval (CI), the computed effect size, and the latter's CI, respectively.

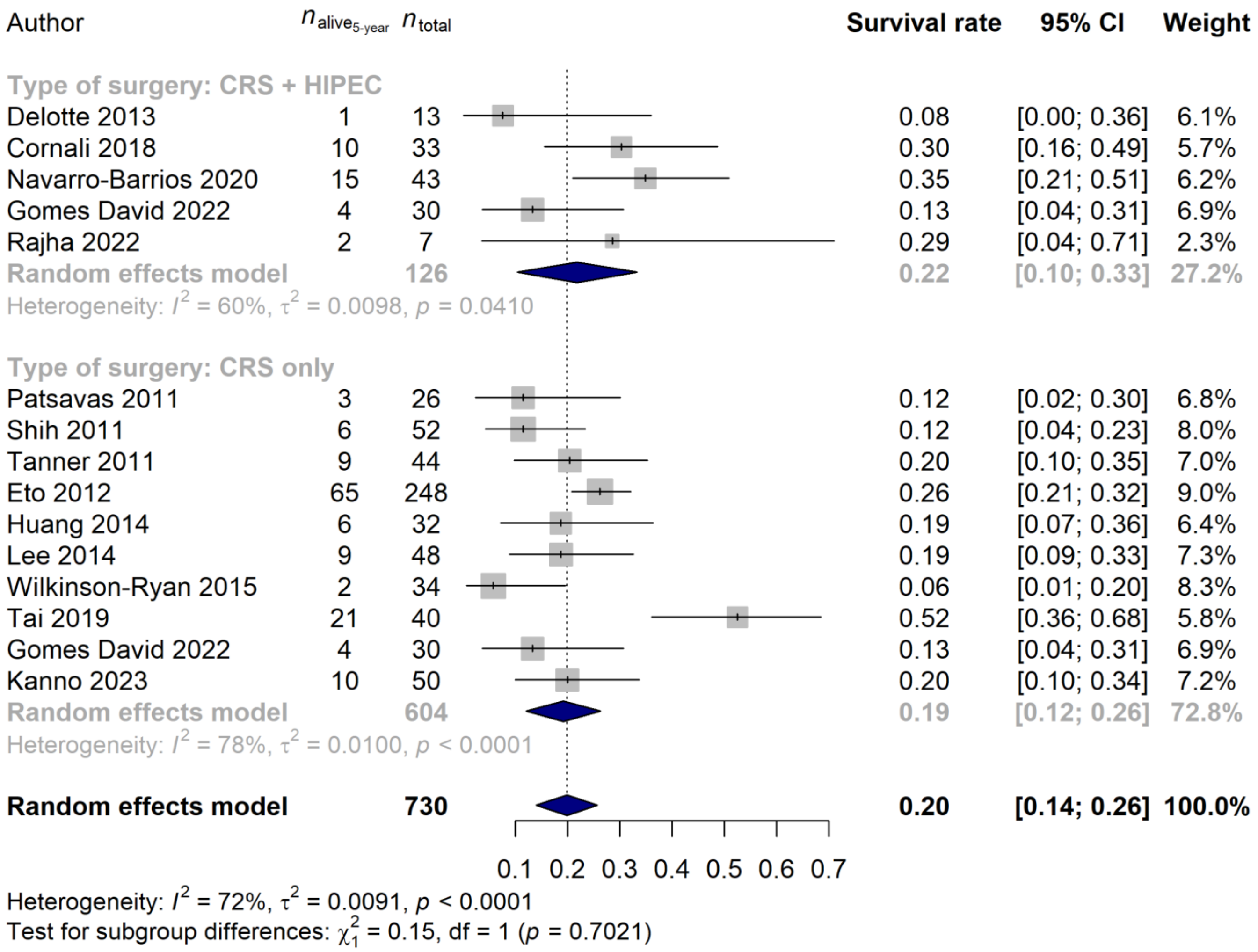

Figure S11. Comparison of the 5-year survival rates of the studies conducted after 2010, which have investigated patients suffering from endometrial cancer with peritoneal metastasis. Patients were treated with cytoreductive surgery (CRS) with or without hyperthermic intraperitoneal chemotherapy (HIPEC). A marginally higher 5-year survival rate is expected favoring CRS + HIPEC. Whiskers, dotted line and blue diamond represents the 5-year survival rate and its 95% confidence interval (CI), the computed effect size, and the latter's CI, respectively.

Table S1. Chemotherapeutic agent and dosage (if provided) details of the selected studies investigating the effect of cytoreductive surgery (CRS) with hyperthermic intraperitoneal chemotherapy (HIPEC) in endometrial cancer with peritoneal metastasis.

Author (Year)	Cases (*n*)	CRS + HIPEC time (hours)	HIPEC time (min)	HIPEC temperature (°C)	HIPEC medication
Helm et al. (2007) [25]	5	9.8	90	43	cisplatin
Bakrin et al. (2010) [8]	5	5.5	90	42	cisplatin (0,7 mg/kg)<br>mitomycin C (0,5mg/kg)
Delotte et al. (2013) [26]	13	5	60	43	cisplatin (50 mg/m^2)<br>doxorubicin (15 mg/m^2)
Abu-Zaid et al. (2014) [27]	6	9.5	90	42	cisplatin (50 mg/m^2)<br>doxorubicin (15 mg/m^2)
Cornali et al. (2018) [28]	33	6.25	60	43	cisplatin (75 mg/m^2)
Navarro-Barrios et al. (2020) [32]	43	7	60 / 90	42	cisplatin<br>paclitaxel
Brind'Amour et al. (2020) [29]	3	9.3	90	42	carboplatin
Chambers et al. (2021) [30]	7	6.9	90	43	cisplatin (100 mg/m^2)<br>paclitaxel (135 – 175 mg/m^2)
Gomes David et al. (2022) [7]	30	6.1	60 / 90	43	cisplatin<br>doxorubicin<br>mitomycin C
Rajha et al. (2022) [31]	7	4.55	60	42	cisplatin<br>doxorubicin

SD: standard deviation.
